# Colorectal Cancer and Mitochondrial Dysfunctions of the Adjunct Adipose Tissues: A Case Study

**DOI:** 10.1155/2018/2169036

**Published:** 2018-11-18

**Authors:** A. P. Burlaka, I. I. Ganusevich, A. V. Vovk, A. A. Burlaka, M. R. Gafurov, S. N. Lukin

**Affiliations:** ^1^R.E. Kavetsky Institute of Experimental Pathology, Oncology and Radiobiology NAS of Ukraine, Kiev, Ukraine; ^2^Ukrainian National Cancer Institute, Kiev, Ukraine; ^3^Kazan Federal University, Kazan, Russia

## Abstract

Excess body weight has been causally linked to an increased risk of different cancer types, including colorectal cancer (CRC) but the mechanisms underlying this association are practically unknown. We investigate redox state-superoxide (SO) generation rate, activity of complex I in electron transport chain (ETC) of mitochondria and of dinitrosyl iron complexes by electron paramagnetic resonance; activity of matrix metalloproteinase (gelatinase) MMP-2 and MMP-9 by gel zymography of adipose tissues (AT) from 46 patients (64.0 ± 1.6 y.o.) with CRC (II–III stages, pT2–3N0–2M0) in the AT adjacent to tumor (ATAT) and at a distance of 3 cm from the tumor (ATD) to follow the connection of the AT redox state with some of the tumor microenvironment indicators. We have incubated the AT species with the tumor necrosis factor *α* (TNF-*α*) to follow its influence on the measured values. As a control, normal AT (NAT) obtained during the liposuction is used. Tumor-induced changes in mitochondrial ETC of ATAT, particularly for Complex I, lead to the enhanced SO generation and consequent oxidative modifications of DNA in ATAT (up to 6.1 times higher than that in NAT and 3.7 times higher than that in ATD, p < 0.05). Gelatinase activity in ATAT is significantly higher than in ATD. A considerable effect of TNF-*α* on ATAT and ATD (but not on NAT, i.e., only on the tissues where the reprogramming of metabolism has already occurred under the influence of tumor) manifested in increase of cellular hypoxia, gelatinase activity, and SO generation rate is observed. The results can be used for better understanding the mechanism(s) of metabolic symbiosis of tumor and AT as well as serving as a basis for new therapeutic approaches.

## 1. Introduction

Obesity is recognized as the second highest risk factor for cancer after tobacco smoking [1–7]. “Obesity-related cancers comprise about 27% of the total global burden of cancer” [2] and “at present impact more populations in highly developed countries, where 67% of all” body mass index (BMI) related cancers are diagnosed [2]. Epidemiological studies show that overweight can account up to 20 % of all cancer-related deaths [3, 4].

“Understanding the link between being overweight or obese and a wide variety of cancers, as well as the biological mechanisms involved, remains an evolving and currently very active area of research” [8]. One of the recent reviews on that topic is paper [9]. “The complex physiological changes that occur with obesity include alterations in the adipose tissue (AT) production of bioactive factors, growth factors, hormones, and reactive oxygen species (ROS) that can impact” the cancer development [10].

It is accepted that “tumor formation (carcinogenesis) is a multistep process involving initiation, promotion, and progression, ultimately leading to a clonal expansion of mutated cells. Diverse chemical and physical agents are able to modulate this complex process resulting in a clinical tumor starting from a single cell” [11]. Oxidative stress thought to be a very central mechanism involved in the process of carcinogenesis and obesity [11–18]. “An imbalance between ROS and the antioxidative capacity of a cell may lead to oxidative damage of cellular macromolecules (DNA, proteins, and lipids) which can result in (i) the formation of mutagenic DNA lesions and (ii) the modulation of intracellular signaling pathways affecting central parameters of the cell, for example, the redox-status, apoptosis, DNA repair mechanisms, and cellular proliferation. DNA bases can be oxidized by ROS which may result in incorrect base pairing leading to mutations” [11].

“Colorectal cancer (CRC) is the third most common cancer in both sex with more than a million of new cases per year and more than 500,000 deaths registered worldwide with few treatment options especially for advanced and metastatic patients” [19]. Different probable mechanisms linking obesity and CRC as well as a brief analysis of the state-of-the-art knowledge gathered so far are reviewed in paper [20]. One of the targets sensitive to damaging influence of CRC tumor in obesity is mitochondria of adipocytes [21]. Mitochondrial respiratory complexes are primary sources of ROS in the cell [22]. The activity of Complex I of electron transport chain (ETC) of mitochondria, the level of dinitrosyl iron complexes, and levels/generation rate of free radicals, like superoxide radical (SO, ${O_{2}}^{\underline{∙}}$), can be accessed by techniques of electron paramagnetic resonance (EPR), also known as electron spin resonance (ESR, see [23–35] as example of the recent publications on this topic, including colorectal cancer [35] affected tissues research).

Like adipose tissue, tumor microenvironment is composed of multiple cell types that favor a proinflammatory and protumorigenic environment [16, 36]. It is known that the levels of proinflammatory cytokines—a serum tumor necrosis factor (tumor necrosis factor *α*, TNF-*α*)—increase in obesity and decrease with weight loss [16–20]. It has been observed that the proinflammatory role of TNF-*α* becomes involved in all stages of tumorigenesis that include tumor cell transformation, survival, proliferation, invasion, angiogenesis, and metastasis. Animal models have shown a positive relationship between TNF-*α* and tumor development and progression in CRC [16].

Long-term studies have revealed that extracellular matrix (ECM) remodeling proteinases, such as matrix metalloproteinases (MMP), are the principal mediators of the alterations observed in the microenvironment during cancer progression [25, 37–40]. Among the members of the MMPs family, gelatinases MMP-2 and MMP-9 are considered to be the essential regulators of the tumor microenvironment [25]. MMP and ROS are regarded as potential diagnostic and prognostic biomarkers in many types and stages of cancer and targets for the therapeutic treatments [17, 37]. Concerning obesity, contrasting results about the relation between the mentioned MMP activity and adiposity are reported [41, 42].

The purpose of this work was to compare the redox status in normal AT (NAT) of individuals without cancer (for whom no cancer was diagnosed) and in AT of patients with CRC adjacent to the tumor (ATAT) and at a distance of 3 cm from it (ATD), activity of matrix metalloproteinase (MMP)-2 and -9 in these tissues, and the impact of TNF-*α* on them. Some preliminary results were published previously [43].

## 2. Materials and Methods

The research was carried out on samples of AT of 46 patients (25 male, 21 female) with CRC at disease stages II-III (pT_2-3_pN_0-2_pM_0_) with the average age 64.1 ± 1.6 years. For 17 patients BMI was in the range 18.5–24.9 (normal weight), and for 29 patients BMI was measured to be ≥ 25.0 (overweight). Histologically the investigated tumors were moderately differentiated (G2, 26 patients) and badly differentiated (G3, 20 patients) adenocarcinomas. As a control, normal adipose tissue (NAT) of 11 conditionally healthy people (6 males, 5 females, BMI > 25.0 for whom no cancer was diagnosed) obtained after liposuction performed at specialized medical center was used. All participants expressed their prior written consent to take part in the research. All procedures followed were in accordance with the ethical standards of the responsible committee on human experimentation (institutional and national) and with the Helsinki Declaration of 1964 and later amendments. The investigated species were stored at liquid nitrogen temperature (T = 77K).

The AT tissues were homogenized by using a chopper mesh of 0.2 mm in diameter. To investigate the effect of TNF-*α* in vitro on adipocyte mitochondria, 1 ml of sodium chloride physiological solution was added to the resulting fat homogenate, which contained 3 *μ*g of TNF-*α* (Sigma-Aldrich, USA), stirred with a glass rod, and incubated for 1 hour in a thermostat at T =36°C. Both inactivated and incubated ones with TNF-*α* species were then studied. Totally 92 (2×46) species of those adjacent to tumor AT (ATAT), 52 (2×26) species taken at distance of 3 cm from the tumor (ATD), and 22 (2×11) AT samples of control group (NAT) were investigated.

Activity of complex I of the mitochondrial ETC and the level of dinitrosyl iron complexes (NO-FeS) were measured as the intensity (amplitude) of the corresponding EPR lines in EPR spectra by using RE-1307 (USSR, Russia) and Bruker ESP-300 (Bruker, Germany) EPR spectrometers at T = 77K [27, 30]. The spectrometers operate at conventional microwave frequency of 9.5 GHz, X-band. Pieces of specifically oriented ruby crystals on the wall of the EPR cavity and reference sample (Mn^2+^ in MgO) were exploited for the quantification of magnetic field values and intensity of EPR signals. Generation rate of ${O_{2}}^{\underline{∙}}$ radicals at room temperature (RT) was measured by spin-trap EPR technique exploiting 1-hydroxy-2,2,6,6-tetramethyl-4-oxo-piperidine hydrochloride (TEMPONE-H) from Sigma-Aldrich as spin traps, correspondingly [30, 44].

Concentrations of matrix metalloproteinase-2 and -9 (MMP-2 and MMP-9) in samples both in active and latent forms were determined by gelatine zymography, the polyacrylamide gel electrophoresis-based method with using sodium dodecyl sulfate (SDS). MMP-2, 9 are considered to be the major MMPs involved in invasion and metastasis of cancer because of their capacity to degrade type IV collagen, an important component of basement membranes. After the gel washing active forms of MMP-2 and MMP-9 were visualized in the form of discolored strips on a blue background, their localization was determined by molecular weight standards (Merck, Germany, 72 and 92 kDa, correspondingly). Proteolytic activity was estimated from the area of clear lysis bands of degraded protein on a uniformly blue background and was expressed in arbitrary units (a.u.). TotalLab 1.01 program tool was used for the calculation and report [28].

Statistical processing of data was provided using variational statistics methods using programs “STATISTICA 8.0” and “Prism 4.0”. The probability of differences between indicators was assessed using Student's t- criterion. The data are presented as mean ± SD (standard deviation). The statistical significance was accepted for p < 0.05.

## 3. Results

**Figure 1:**
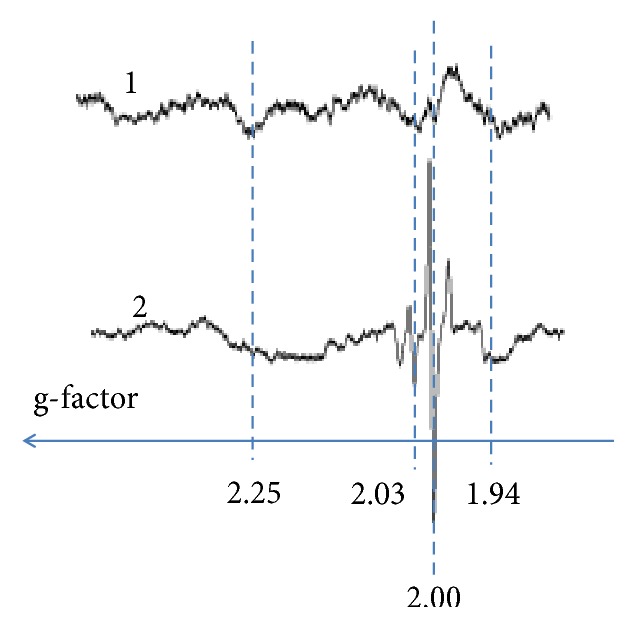
Examples of EPR spectra recalculated to the units of the spectroscopic g-factor for (1) normal adipose tissue (NAT) and (2) adipose tissue from CRC patients at disease stages II-III (ATAT).

**Figure 2:**
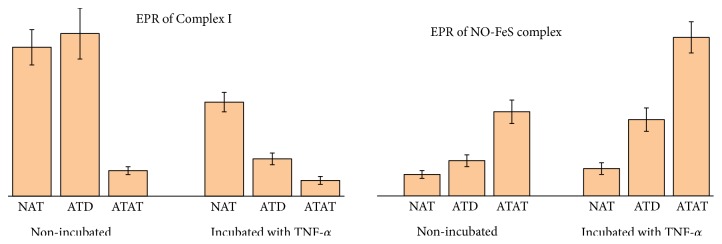
Left pane: activity of Complex I of mitochondrial ETC in cells of NAT, ATD, and ATAT before and after the incubation with TNF-*α*. Right panel: level of NO-FeS-proteins complexes in ETC of mitochondria in cells of NAT, ATD, and ATAT before and after the incubation with TNF-*α*. Data presented as Mean ± SD. Numerical values are given in Table 1.

**Figure 3:**
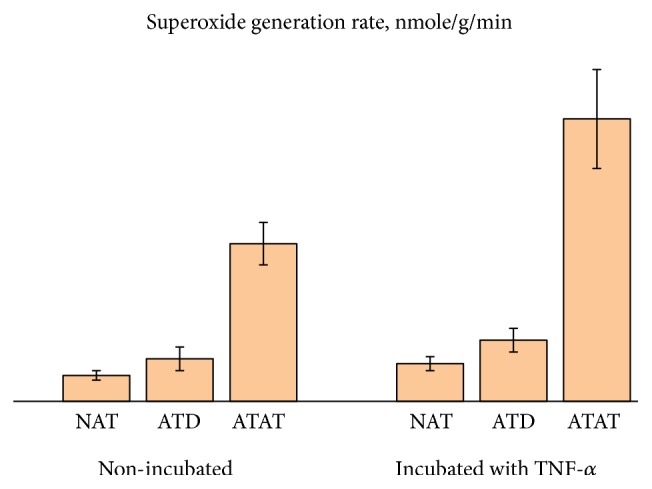
Superoxide generation rate by mitochondria of NAT, ATD, and ATAT before and after the incubation with TNF-*α*. Data presented as mean ± SD. Numerical values are given in Table 1.

Typical EPR spectra for NAT and ATAT are shown in Figure 1. In NAT and ATAT the following signals could be registered [30]: (1) with g ≈ 1.94 from the iron-sulfur (FeS) cluster N2 of NADH: ubiquinone oxidoreductase, also called respiratory complex I of ETC; (2) from “the “free” radical centers practically completely localized in mitochondria, semiquinones of flavoproteins found in the inner membrane of mitochondria and coenzyme Q semiquinones (ubisemiquinones) at g ≈ 2 .00; (3) signal at g ≈ 2.03 due to the formation of NO and N types FeS-protein complexes; (4) signal with g ≈ 2.25 related to the activity of the cytochrome P-450. As a rule, in ATAT of CRC patients at stages II-III signals at g ≈ 2 .00 and g ≈ 2.03 grow; signal at g ≈ 2.25 becomes less. The results confirm the metabolic changes (reprogramming) in mitochondria with a shift towards glycolysis. Those extracted from the EPR measurements data for some of the intrinsic and trapped (superoxide) paramagnetic centers are presented in Table 1; Figures 2 and 3.

**Figure 4:**
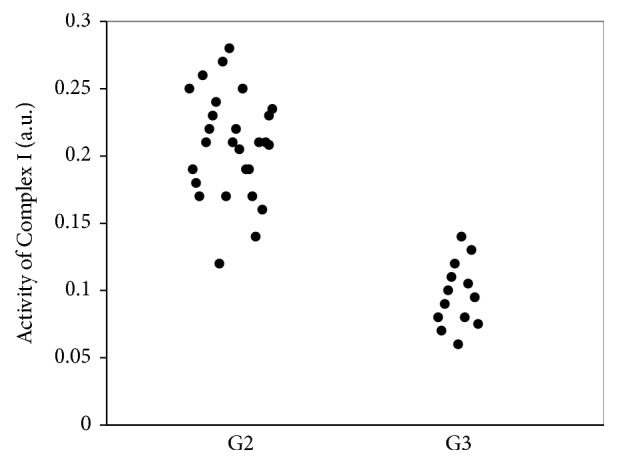
Activity of Complex I of mitochondrial ETC in species of ATAT (before the incubation with TNF-*α*) on differentiation grade G.

We found that the reduction of activity of Complex I of ETC of mitochondria in ATAT depends on the degree of differentiation of the tumor (Figure 4). The degree of differentiation of CRC G2 corresponds to the level of activity of FeS-proteins 0.22±0.04 a.u. and G3 – to 0.10±0.02 a.u. The activity level of Complex I of ETC of mitochondria in ATAT correlates with the degree of differentiation of CRC (r=0.64; r=0.47; p<0.05).

**Figure 5:**
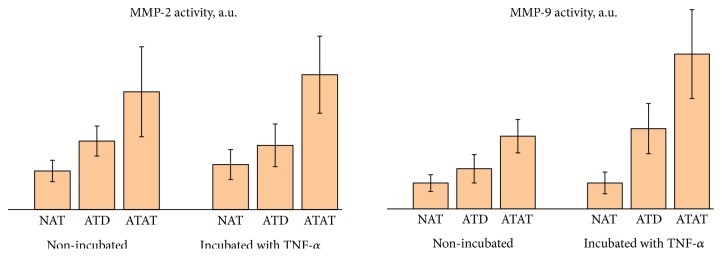
Left panel: activity of MMP-2; right panel: activity of MMP-9 in NAT, ATD, and ATAT before and after the incubation with TNF-*α*. Numerical values are given in Table 1.

Activity levels of MMP-2 and MMP-9 in the NAT, ATD, and ATAT tissues, nonincubated and incubated with TNF-*α*, are listed in Table 1 and shown in Figure 5.

## 4. Discussion

From the presented results it follows that redox state of tumor defines the changes of the cell functioning not only in adjacent but also in the remote adipose tissue. ${O_{2}}^{\underline{∙}}$ generation rates in mitochondria of adipocytes in ATD are higher than those in norm while in adjacent to tumor AT they are almost 4 times higher than those in norm (p < 0.05). This indicates a damaging effect of tumor on ETC. Under the influence of TNF-*α*, superoxide generation rate in ATAT becomes significantly higher (in 1.8 times compared to the nonincubated tissue, 4.6 times and 7.5 times compared to the incubated ATD and NAT, correspondingly).Therefore, the regulatory effect of cytokine production on ETC in ATAT is detected, an increase in the electron-oxygen interaction that leads to the system response to inflammation valued in the growth of superoxide generation.

Comparison of levels of NO-FeS complexes in ATAT and NAT shows that the damaging effect of CRC on mitochondrial ETC is manifested in the increase of levels of these complexes up to 4 times (Table 1). TNF-*α* further significantly increases this value not only in ATAT but also in ATD, up to 2 times. In NAT during the incubation with TNF-*α*, the specified index remains almost unchanged (p > 0.05) that may indicate the possibility of realization of the TNF-*α* effects only for the reprogrammed ETC of mitochondria of adipocytes (which are already involved in the process of tumor growth), that is, adipocytes of ATAT and ATD.

Activity of Complex I of mitochondrial ETC in NAT and ATD (Table 1) is 5.8 and 6.4 (p < 0.05) times, respectively, higher than that in ATAT. Incubation of samples with TNF-*α* in vitro induces significant decrease in levels of activity of Complex I ETC compared especially in ATD (4.2 times, p < 0.05). Some data allow suggesting that two subunits of Complex I of ETC of mitochondria, NDUFA4 and NDUFA5, determine its electron transport function and, thus, form the redox status of tumor that correlates with its metastatic potential which may have an important prognostic significance [21]. Consequently, it can be supposed that exactly in these subunits the mitochondrial Complex I of ETC of AT cells is damaged by the inflammatory cytokines, in particular, by low concentrations of TNF-*α* (in concentrations that do not cause the cell death), which could result in lowering the activity of FeS-proteins in another subunit, NDUFA13. It is known that NDUFA13 subunit is the first one to be reprogrammed from the oxidative metabolism to glycolysis during tumor progression [21].

Data for the gelatinases activity depending on the presence of tumor and under the influence of TNF-*α* are also presented in Table 1. The activity of MMP-2 and MMP-9 in ATAT was found to be about 2 times higher than that in ATD and about 3.0 times higher than that in NAT. Thus, the tumor enhances the activity of gelatinases and, probably, the level of ECM destruction in ATAT. Reprogrammed adipocytes of ATAT, as shown above, produce high levels of superoxide radicals which stimulate inflammation and regulate the synthesis and activation of gelatinases [45].

TNF-*α* significantly enhances the activity of MMP-9 in ATD and ATAT (about 2 times) but has no effect on NAT (p > 0.05, Table 1). However, the activity of MMP-2 in NAT and ATD remained almost unchanged while in ATAT it only slightly increases compared to the nonincubated samples (p > 0.05). These results can be explained by redox-dependent activation of MMP-9 that can be confirmed by correlation between the superoxide generation rate by adipocytes and activity of MMP-9 in adipose tissues exposed to TNF-*α* (r = 0.53; p<0.05) obtained in the present investigation. A number of studies have established that TNF-*α* regulates the activity of proangiogenic MMP-9 through MAPKs signaling pathway [46], activation of NF-*κ*B [47], proteolytic modeling of receptor of cyclooxygenase-2 [48], and other pathways with ${O_{2}}^{\underline{∙}}$ radicals as mediators [45]. MMP-2 is also a redox-dependent enzyme but to date there is little information about its regulation by TNF-*α* [46] while some data exist demonstrating that MMP-2 could, in its turn, regulate TNF-*α*, correcting the imbalance of proinflammatory factor according to the levels of destruction of ECM and preventing, therefore, the development of inflammation [49]. Absence of the changes in the activity of MMP-2 under the action of TNF-*α* may be a consequence of mutual regulation of MMP-2 and TNF-*α*: the excessive concentrations of proinflammatory factor start the relevant compensatory mechanisms, particularly, through the regulatory influence of MMP-2.

## 5. Conclusions

Our results confirm that mitochondria of AT play an important role in energy metabolism and may be damaged by tumor. In addition, in the cells of AT of patients with malignant tumors of gastrointestinal tract we have revealed a dysfunction of the mitochondrial electron transport Complex I, change of the structure of ETC of mitochondria manifested in the modification of redox state of adipose tissues. The damage of the oxidative phosphorylation causes the relevant changes in the cellular redox state and initiates the generation of superoxide radicals. Dysfunction of mitochondria of adipocytes can initiate dysplasia and be used in the diagnosis and prognosis of the disease, which correlates with indicators of metastasis and aggressive phenotype of CRC.

The incubation of NAT, ATD, and ATAT with the proinflammatory cytokine TNF-*α* leads to the changes in redox state of mitochondria and activation of a number of the measured factors of inflammation only in tissues where the reprogramming of metabolism has already occurred under the influence of tumor, in ATAT and to a lesser degree in ATD.

Additionally, the data obtained show that electron paramagnetic resonance could be productively used to study the crosstalk between adipose tissue and tumor as well as for the evaluation of the effectiveness of the therapy. The presented approach could serve as an initial step to the implication of EPR imaging (EPRI) for the cancer-obesity related research and clinical implication.

## Figures and Tables

**Table 1:** Superoxide generation rate, levels of NO-FeS, Complex I, and gelatinase activity in ATAT, ATD, and NAT before and after incubation with TNF-*α*. Data are presented as mean ± SD.

	ATAT (n = 46)		ATD (n = 26)		NAT (n = 11)	
	Before incubation	After incubation	Before incubation	After incubation	Before incubation	After incubation

${O_{2}}^{\underline{∙}}$, nmole/g·min	0.67±0.09^#^	1.20±0.21^#,*∗*^	0.18±0.03^#^	0.26±0.05	0.11±0.02	0.16±0.03

NO-Fes complex, a.u.	0.43 ± 0.06^#^	0.81±0.08^#^	0.18±0.05^#^	0.39±0.06	0.11±0.02	0.14 ± 0.03

Complex I of ETC	0.13±0.02*∗*	0.08±0.02^#,*∗*^	0.83±0.13	0.19±0.03^#,*∗*^	0.76±0.09	0.48±0.05*∗*

MMP-2, a.u.	5.5±2.1^#^	6.3±1.8^#,*∗*^	3.2±0.7^#^	3.0±1.0	1.8±0.5	2.1±0.7

MMP-9, a.u.	8.7±2.0^#^	18.5±5.3^#,*∗*^	4.8±1.7	9.6±3.0^#,*∗*^	3.1±1.0	3.1±1.3

Note: ^#^ p < 0.05 compared to nonincubated NAT; ^*∗*^ p < 0.05 compared to the incubated NAT.

## Data Availability

The data used to support the findings of this study are available from the corresponding author upon request.
